# Devitalization of Dental Pulp

**Published:** 1894-05

**Authors:** A. W. Diack

**Affiliations:** Detroit, Mich.


### Devitalization of Dental Pulp.

BY A. W. DIACK, D.D.S., DETROIT, MICH.

Read before the Detroit Dental Society, March 12, 1894.

Among the many obstacles that have presented themselves to me, and which have a tendency to induce worry and anxiety, since I have been in practice, not the least has been the discovery of exposed pulps. They are to me a precursor of subsequent trouble; for either they require a great amount of nervous and skillful work or else the patient will lose all faith in the so-called modern methods and painless dentistry of recent years.

Without entering into a discussion concerning the advisability of conservative treatment, but passing on to that condition where we are resolved that the pulp shall be killed, I will consider the subject of the "Devitalization of Pulps," and I will aim, so far as possible, to bring forth some method, by discussion, which will be more for the patient's comfort than the one which is now employed by the profession generally.

There are two things to be attained in this operation before we, as dentists, and the public, as patients, are to be thoroughly satisfied; that is, before we can have a painless devitalization.

The first is the rapidity of the devitalization, and the second is either a new agent for the purpose or else a new method of manipulating the old.

These divisions, as you can see, are merely arbitrary and are really mutually dependent on each other; for I am a firm believer in the fact that dental patients suffer more in the anticipation than realization, and that if we cause a patient to come several times and at each sitting do some painful work, we shall find that the last sitting is always the worst, and that the patient acts in a more nervous manner. So I say that if we succeed in shortening the period of devitalization we have taken a step toward making the operation painless.

To pass on now to the agents used: Formerly, there were several methods in vogue, among them the operative procedure of removing the pulp without the previous application of a medicinal agent. Even, now, I have heard and read of those advocating this method, some saying that they never resort to the ordinary means. To my mind this is only a relic of barbarism, or else my opinion is based upon my own lack of ability.

Among the medicinal agents used, were the capping with the oxychloride and oxyphosphate of zinc, prolonged applications of carbolic acid, creasote, etc. But these have generally been allowed to pass into disuse, owing to the long time necessary to accomplish the result and the great uncertainty of the action. Sufficient to say, that the agent which is pre-eminent in this branch is arsenious acid. It was first used, I believe, some fifty years ago, and has held its own against all other drugs ever since. Several theories have been advanced to account for its caustic action upon the dental pulp, but I will not enter upon that at all, other than to mention the fact that it caused in many instances great nervous irritation and pain. It is this physiological effect which we must overcome if we would have a painless operation. Several agents have been combined with the arsenic to overcome this, but as yet to my knowledge there has been nothing which accomplishes the result.

The ordinary application consists of equal parts of arsenious acid and sulphate or muriate of morphine, made into a paste by the addition of creasote or carbolic acid. Other preparations have contained other agents instead of the morphine. One preparation, the formula of which is a secret, is spoken of highly by its users.

My method of using this application, which I think is the method in general use, is to clean the cavity of decay as thoroughly as possible and expose the pulp, so that there will be free access to the blood vessels, and if possible cause a few drops of blood to flow from the pulp. Then about one-sixtieth of a grain of the arsenic in combination with the morphine is spread gently over the exposure. A small piece of loose cotton is then laid over the application, and the final piece of cotton moistened with sandarac or chloro-percha previously warmed is placed so as to prevent pressure upon the pulp. The cavity, of course, is kept free from moisture, either by the rubber cloth or the napkin. The patient is then discharged for twenty-four or forty-eight hours, and at that sitting the subsequent operation is begun, if all is well. If not, as I can easily tell by the sensitiveness of the pulp, I repeat the application as before, except that I sometimes allow seventy-two hours to intervene before the next sitting. Even then the pulp in some cases seems unaffected. These cases are then given a prolonged treatment dependent upon circumstances.

Now, with this method, in the majority of cases, I find considerable pain even though I have taken special care in making the application. I have tried in several ways to overcome this pain, both by the use of local means and systemic analgesies. Systemically I have used acetanilide and phenacetine, the former in the form of tablets of the acetanilide compound, which contains:

| | | |
|---|---|---|
| Acetanilide, | - - - | gr. ij |
| Camph-Monobrom, | | |
| Caffeine Citrate, | - - | aa gr. ss |

Phenacetine I have used in doses of ten grains to be followed in an hour by five grains in a teaspoonful of brandy.

To say that these have been successful would be exaggeration, for contrary to the observations of other men, I have found them of doubtful efficacy. To illustrate, a young physician presented an upper right first molar; the nerve had been in a state of chronic inflammation for several years, as was shown by pain at irregular intervals, and an uncomfortable feeling in the tooth. This condition was the result of having a large metal filling almost directly in contact with the pulp. I exposed the pulp and made the application in the ordinary manner. The patient suffered very much, and I prescribed phenacetine in ten grain doses. He reported the next day, as I told him, and said that he continued to have more or less pain until the following morning, although he had taken other anodynes than the phenacetine. He had kept an account of what he had taken, and I found that he had taken: thirty grains of phenacetine, twelve grains of acetanilide, together with the camphor and caffeine, and three-quarters of a grain of codeine.

This has a tendency to make a person rather skeptical regarding the good to be obtained from systemic drugs.

As a local sedative, for the devitalization of dental pulps, I have used several agents in the place of the morphine, which drug I believe has very little anodyne properties, tannic acid, aristol, iodoform, hydrochlorate of cocaine and oil of cloves; all have been used in the mixture but with negative results, at least in my hands. Recently, however, I have been using a mixture of belladonna extract and arsenious acid, in proportion of two of the extract to one of the acid. This is made into a paste with glycerine.

I use the extract of belladonna because of the well known anodyne effect of the drug on the sensory nerves. The glycerine I use because of its emollient property and also because of its power of being easily absorbed. I have used this formula in several cases, and, so far, feel very hopeful, as I have yet to hear of any severe pain. But it is altogether too soon to even predict any decided results. Perhaps at some future time I will be able to praise the formula in stronger terms, unless I become disgusted with it as I have with the others.

I came across a case, not long ago, which puzzled me considerably, and which I think will shed some light upon this subject.

A young man aged twenty-three, of good health, presented a lower left first molar, the crown of which was about one-third destroyed by caries. The dentine was covered with that decay of the reddish-brown, leathery character, which is seen so frequently in these cases. Patient never had any pain in the tooth previous to that time, but then he was suffering very much. I made a diagnosis of pericementitis, caused by death of pulp, and opened into the pulp chamber. When the entrance was effected a discharge of pus amounting to four or five drops took place; this was soon followed by blood. Pain was temporarily relieved, but soon reappeared. I then made the entrance to the chamber as large as possible, but was unable to accomplish much, as the pulp was extremely sensitive. I treated the pulp to allay pain and reduce inflammation, and told the patient to return the next day. He had slight pain during the interim. At the second sitting the pulp could not be touched, being so sensitive. I made an arsenical application and asked the patient to return next day. This was done, but no diminution of vitality was appreciable.

The application was repeated at least four times, but with no results. I finally used hydrochlorate of cocaine in crystals, and removed part of the pulp, but even this ceased to have an effect. At this stage the patient and myself both being discouraged I extracted the tooth. A curious condition existed in this case, which I think accounts for the lack of any action of the arsenic or cocaine. The color of the pulp, instead of being of that reddish shade, was pale and colorless, and after the first sitting there was no hemorrhage at all. It seemed as though the blood vessels were entirely devoid of function, while the nerves of the pulp retained their functions in an exalted degree. This explains why the medicines were not absorbed; and I believe this is a condition which exists to a greater or less degree in those cases where repeated applications are necessary.

Now, gentlemen, to some of you it may seem that I am making a mountain out of a mole hill, in taking this subject, but I have felt justified, and no doubt will again feel justified when I hear the complaints of patients when the subject of "nerve-killing" is broached. I am convinced that in a great majority of cases the operation is more to the patient than we think. It means in some instances so much of pain that the patient is very unwilling to undergo a repetition of it. It is not only my own patients who complain, but those of other men also, so I know that all of this trouble cannot be ascribed to lack of ability, as some of you may think from the fuss I have made in this paper. However, my chief end in writing this was to get some new light on the subject, and I hope that the discussion will shed some.

---

Cocaine May Be Tested for Thus: Add to the solution to be examined a drop of a solution of potassium bichromate. If cocaine be present a precipitate will form which vanishes rapidly, and on warming, the liquid turns green and gives off fumes having a peculiar odor—that of benzoic acid.—*The Literary Digest.*

